# Corrigendum: A magneto-electro-optical effect in a plasmonic nanowire material

**DOI:** 10.1038/ncomms14497

**Published:** 2017-02-07

**Authors:** João Valente, Jun-Yu Ou, Eric Plum, Ian J. Youngs, Nikolay I. Zheludev

Nature Communications 6: Article number: 7021; DOI: 10.1038/ncomms8021 (2015); Published 04 24 2015; Updated 02 07 2017

This Article was originally published under a CC BY-NC-ND 4.0 license, but has now been made available under a CC BY 4.0 license. The PDF and HTML versions of the paper have been modified accordingly.

